# Explainable AI in radiology: a white paper of the Italian Society of Medical and Interventional Radiology

**DOI:** 10.1007/s11547-023-01634-5

**Published:** 2023-05-08

**Authors:** Emanuele Neri, Gayane Aghakhanyan, Marta Zerunian, Nicoletta Gandolfo, Roberto Grassi, Vittorio Miele, Andrea Giovagnoni, Andrea Laghi, SIRM expert group on Artificial Intelligence

**Affiliations:** 1 Academic Radiology, Department of Translational Research and of New Surgical and Medical Technology, University of Pisa, Pisa, Italy; 2 Medical-Surgical Sciences and Translational Medicine, Sapienza University of Rome, Sant’Andrea Hospital, Rome, Italy; 3 Diagnostic Imaging Department, VillaScassi Hospital-ASL 3, Corso Scassi 1, Genoa, Italy; 4 Radiology Unit, Università Degli Studi Della Campania Luigi Vanvitelli, Naples, Italy; 5 Department of Radiology, Careggi University Hospital, Florence, Italy; 6 Department of Radiological Sciences, Radiology Clinic, Azienda Ospedaliera Universitaria, Ospedali Riuniti Di Ancona, Ancona, Italy

**Keywords:** Artificial intelligence, Black-box problem, Explainable AI, Trustable AI

## Abstract

The term Explainable Artificial Intelligence (xAI) groups together the scientific body of knowledge developed while searching for methods to explain the inner logic behind the AI algorithm and the model inference based on knowledge-based interpretability. The xAI is now generally recognized as a core area of AI. A variety of xAI methods currently are available to researchers; nonetheless, the comprehensive classification of the xAI methods is still lacking. In addition, there is no consensus among the researchers with regards to what an explanation exactly is and which are salient properties that must be considered to make it understandable for every end-user. The SIRM introduces an xAI-white paper, which is intended to aid Radiologists, medical practitioners, and scientists in the understanding an emerging field of xAI, the black-box problem behind the success of the AI, the xAI methods to unveil the black-box into a glass-box, the role, and responsibilities of the Radiologists for appropriate use of the AI-technology. Due to the rapidly changing and evolution of AI, a definitive conclusion or solution is far away from being defined. However, one of our greatest responsibilities is to keep up with the change in a critical manner. In fact, ignoring and discrediting the advent of AI a priori will not curb its use but could result in its application without awareness. Therefore, learning and increasing our knowledge about this very important technological change will allow us to put AI at our service and at the service of the patients in a conscious way, pushing this paradigm shift as far as it will benefit us.

## Introduction

In recent years, artificial intelligence has rapidly entered diagnostic imaging, demonstrating a lot of potential, both as a catalyst of the workflow and as an aid to the interpretation of bio-images, becoming a promising engine of the decision support systems in radiology [1]. One of the major drivers behind the steady blossoming of AI in medical imaging is powered not only by the widespread availability of large data sets and advancements in both hardware and software systems, but the urge to achieve greater efficiency in clinical care and management. By providing quantitative image data with radiomics in combination with AI tools, AI in radiology smoothly embeds the essence of diagnostic, predictive, and prognostic applications [2]. The popular pillars for the key AI technologies shaping the future of radiologists cover image processing, computer vision, natural language processing, and much more [3]. Besides, the growing evidence indicates that AI algorithms provide support at all levels of radiology workflow management for a variety of non-diagnostic applications, such as quality, safety, and operational efficiency [1]. The integration of AI into the imaging workflow has the potential to enhance efficiency, minimize errors, and meet specific goals with minimal human intervention. [4]. However, due to the “black-box” nature of AI models, they are often perceived as being less trustworthy by physicians, which has limited their implementation in real-world clinical settings. [5]. To address this issue, the field of Explainable Artificial Intelligence (xAI) has been developed, with the goal of improving the interpretability of AI decisions. The focus of xAI is to create new techniques and algorithms that increase the transparency of the decisions accepted by algorithms and predictive models, thus the reliability and the impact of each feature on the outcome. [6].

This white paper of the Italian Society of Medical and Interventional Radiology (SIRM) is intended to aid radiologists, medical practitioners, and scientists in understanding an emerging field of xAI, enhancing awareness of the black-box problem behind the success of AI, increasing the knowledge of the xAI methods that enable to unveil the black-box into a glass-box, raising consciousness about the role, and the responsibilities of the radiologists for appropriate use of the AI-technology.

### The clinical use of AI and the problem of the black-box

Currently, two primary AI methods are commonly employed in radiology. The first one adopts handcrafted engineered attributes, such as radiomics features, that are used as inputs in cutting-edge machine learning models trained to perform various clinical decision-making tasks [7]. The second method, based on deep neural networks or deep learning (DL), gained significant attention in the last decade [8, 9].

**Fig. 1:**
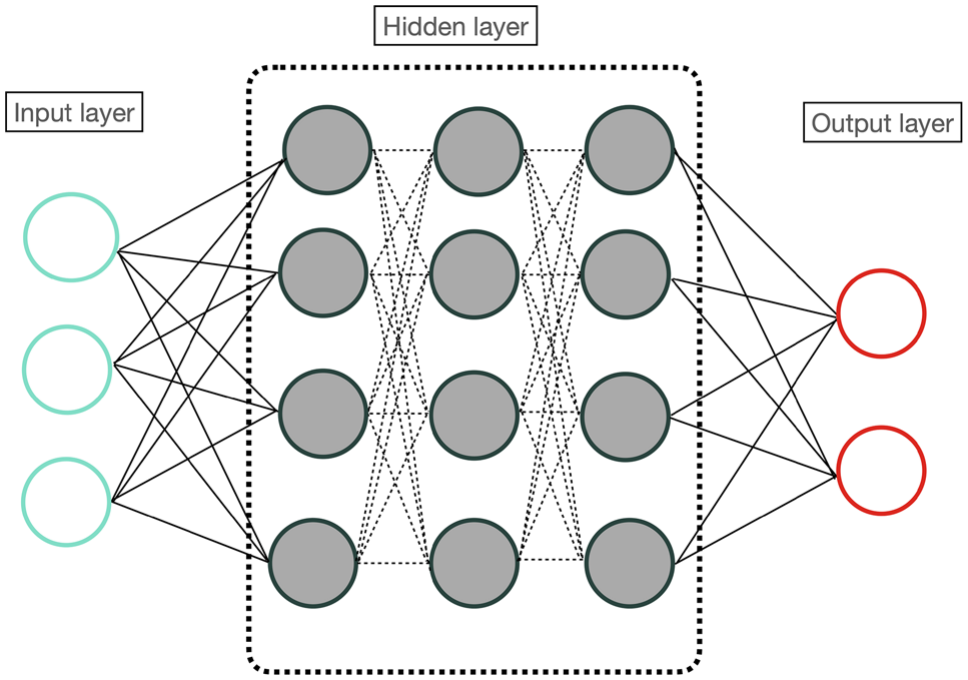
The architecture of the deep neural network consisting of the input layer, the hidden layer and the output layer

There are three primary types of machine learning algorithms including supervised learning, unsupervised learning, and reinforcement learning. In supervised learning algorithms, such as linear and multivariate regression, logistic regression, Naive Bayes, decision trees, k-nearest neighbor and linear discriminant analysis, the input data is labeled. In comparison to supervised learning, the unsupervised learning does not required labeled data. Clustering analysis, anomaly detection, hierarchical clustering, and principal component analysis represent unsupervised learning algorithms. Reinforcement learning is a more advanced machine learning algorithm that solves multi-level problems through learning [7]. DL is a relatively new area of study. While machine learning techniques rely on statistical methods to recognize patterns, DL resembles the human brain and it is best known for its neural network models. A deep neural network typically consists of three types of layers: the Input Layer, the Hidden Layer, and the Output Layer (Fig. 1).

The Input Layer receives the input data, while the Hidden Layer performs various computations on that data. The Output Layer produces the final result. It is important to note that a neural network can have multiple hidden layers, allowing for more complex computations and predictions. One of the advantages of DL algorithms is their ability to learn characteristic attributes from data automatically, with no requirement for human experts to define them beforehand. With sufficient amounts of example data, DL models can identify abnormalities in tissue and avoid the need for human-defined segmentations, which allows for more abstract feature definitions and improves generalizability. DL's ability to learn complex data representations often makes it vigorous against unwelcome variations, including, for example, inter-reader variability, and further enables it to put into a wide range of clinical conditions and frameworks [7]. Table 1 summarizes the main advantages and disadvantages of machine learning and DL methods (Table 1).

**Table 1:** Advantages and disadvantages of machine learning and deep learning

Advantages of machine learning	
Pattern identification	ML analyses large amounts of data discerning patterns that may not be visible to humans
Automation	ML makes predictions and improve algorithms without the need for human intervention
Improvement	ML algorithms able to gain experience improving the accuracy
Multi-dimensional data	ML algorithms can handle complex data
Applications	ML can be applied to a variety of fields in healthcare, including radiology
*Advantages of deep learning*	
Unstructured data	DL models can process unstructured data
Better accuracy	DL models can achieve higher accuracy compared to traditional machine learning models
Automatic feature extraction	DL models automatically learn features, hence avoided manual feature engineering
End-to-end learning	DL models learn to perform a task from input to output, bypassing the need of the intermediate steps
Generalization	DL models can be generalized to unseen data
*Disadvantages of machine learning*	
Data Acquisition	ML requires large and high-quality datasets
Resources	ML needs time and resources to develop algorithms
Interpretation	Interpreting the results generated by ML algorithms can be challenging
High Error-susceptibility	ML is susceptible to errors, especially if the training data sets are biased
*Disadvantages of deep learning*	
Large datasets	DL models require huge amount of data to train
Computationally expensive	DL models are computationally expensive
Difficult to interpret	DL models are often considered black boxes
Overfitting	DL models can easily overfit to the training data, resulting in poor performance on new data
Lack of transparency	DL models can be difficult to debug when they fail due to a lack of transparency

The DL tools can generate extremely reliable outcomes, yet they own an intrinsic “opacity”, and although not entirely opaque, their behavior can be difficult to comprehend. Even experts at the highest level may struggle to fully understand the so-called “black-box” models, the reasonability through which models come to forecasting decisions in areas that are critical and relevant to our society, including healthcare information technology and medical imaging, may be still difficult [10]. The highly opaque nature or inexplicability of AI represents the main element of distrust on the part of medical professionals and patients towards this new technology [11]. This fact generates an obstruction to its practical application, which is particularly reflected in those susceptible fields, where automation influences the existence and survival of the human being, as in a particular way in the sector of healthcare. Applying AI to the field of medicine poses significant challenges. Medical decision-making typically involves uncertainty, incomplete and noisy data sets, and a high level of complexity [12]. As a result, transparency in AI models is particularly crucial in medical care, because of its inner ambiguous quality. While humans may not always be able to explain their reasoning, understanding how an AI model makes decisions can provide confidence in human–machine interactions [13]. With an increasing focus on incorporating ethical standards into AI technology design and implementation, there is a growing demand for “Trustable AI,” a term that with slight conceptual modification may encompass *Valid AI*, *Responsible AI*, *Privacy-Preserving AI*, and *Explainable AI* (xAI). In this context, the xAI aims to display cardinal issues about the decision-making process either for human or machine positions [10].

### What does explainable AI mean?

The xAI is an emerging field with several new strategies and multiple ongoing studies that generate a significant impact on the development of AI in many different areas. Van Lent et al., put in place, first, the concept of xAI by describing their system's ability to explain AI-based predictions [14]. Although the term has been inconsistently applied, it generally refers to a class of systems that can shed light on how an AI system arrives at its settlements [15]. The xAI investigates the reasoning behind the decision-making process, outlines the system's strengths and weaknesses, and predicts the future conduct of the model [10].

Thus far, the xAI may be considered an umbrella term covering certain aspects of xAI [10, 16], including

- *Interpretability,* refers to the understanding of the output of the algorithm for end-user implementation
- *Explainability*, involves clarifying how a decision was reached so that a broader range of users can understand it.
- *Transparency*, refers to the degree of the incomprehensibility of the model.
- *Justifiability*, involves providing an in-depth case to support certain conclusions.
- *Contestability*, relates to the fact that users are able to proclaim a particular decision.

In AI, there is often a negative association between the complexity or depth of a system and its interpretability. This inherent tension between predictive accuracy and explainability frequently results in the most accurate methods (such as DL) being the least transparent, while the most interpretable methods (like decision trees) are less accurate [17]. It is essential to attain a balance between the performance of the model and its interpretability, as the first concept will markedly improve patient care, while the second one will enhance the adoption and trust of AI in radiological practice [16].

***Ethical, legal, and social issues (ELSI) of xAI***

The pursuit of transparent and explainable AI in recent years has not only sparked significant research efforts in the field, but it has also become a central focus of many ethical and responsible design proposals [5, 11, 18]. Additionally, people often express concerns about privacy and security when it comes to AI technologies [19]. The need for greater clarity and transparency was recognized by various institutions. The European Commission has produced a white paper aimed at creating a regulatory framework for a digital ecosystem of trust in reliable AI, among which the fundamental ethical requirements identified are transparency and explainability. In the Ethical Guidelines for reliable AI document, drawn up by the High-Level Expert Group on AI of the European Union, the right is stated to “require an adequate explanation of the decision-making process” whenever AI “significantly affects the people's lives “ [20].

It is intrinsic that after human intelligence fails with significant consequences, the appropriate best practice is to find the root causes, make improvements, and learn from our own mistakes. In the case, the AI fails, it is important to acknowledge it, and increasingly, there is a demand for an explanation of what went wrong in the AI decision-making algorithm [21]. The practical outcome is to establish accountability both in the legal and social sense. Without a clear assignment of liability, it is unlikely that AI can be widely implemented in real-world situations. Therefore, an unforeseen legal challenge may arise, which could have significant implications. [21]. However, addressing only ethical or legal concerns surrounding AI may not be sufficient. All Ethical, Legal, and Social Issues (ELSI) of AI deserve equal attention and certainly should be ahead of AI and xAI implementation in healthcare, as the aim of an ELSI reflection is to provide decision-makers and stakeholders with a comprehensive understanding of the ethical, legal, and social issues associated with a particular technology or practice [22].

In recent times, explaining the output of AI systems has become a crucial issue, not just technically but also legally and politically. There is a general belief that explainable AI systems should be ethically desirable and possibly even legally necessary, which has driven much research in this area [23]. The question of transparency has been given significant attention in regulatory proposals at the EU level, particularly in the proposed Artificial Intelligence Act (AIA). However, discussions and consultations around regulating AI systems are ongoing, and the obligations for explainability under existing regulations and future policies are still being debated [18].

### Solutions to the black box?—explainable AI models

Explainability methods, either in the research setting or legal communities, are being recommended as a practical means to increase transparency and discrimination in AI models [24].

A few proposals to classify the xAI techniques have been promoted so far based on the three fundamental dimensions [25]:

- the xAI technique implementation stage (ante-hoc, post-hoc)
- the xAI technique is intended to provide either a global explanation of the model or a local explanation of a prediction
- the xAI technique is model-specific or model-agnostic

**Fig. 2:**
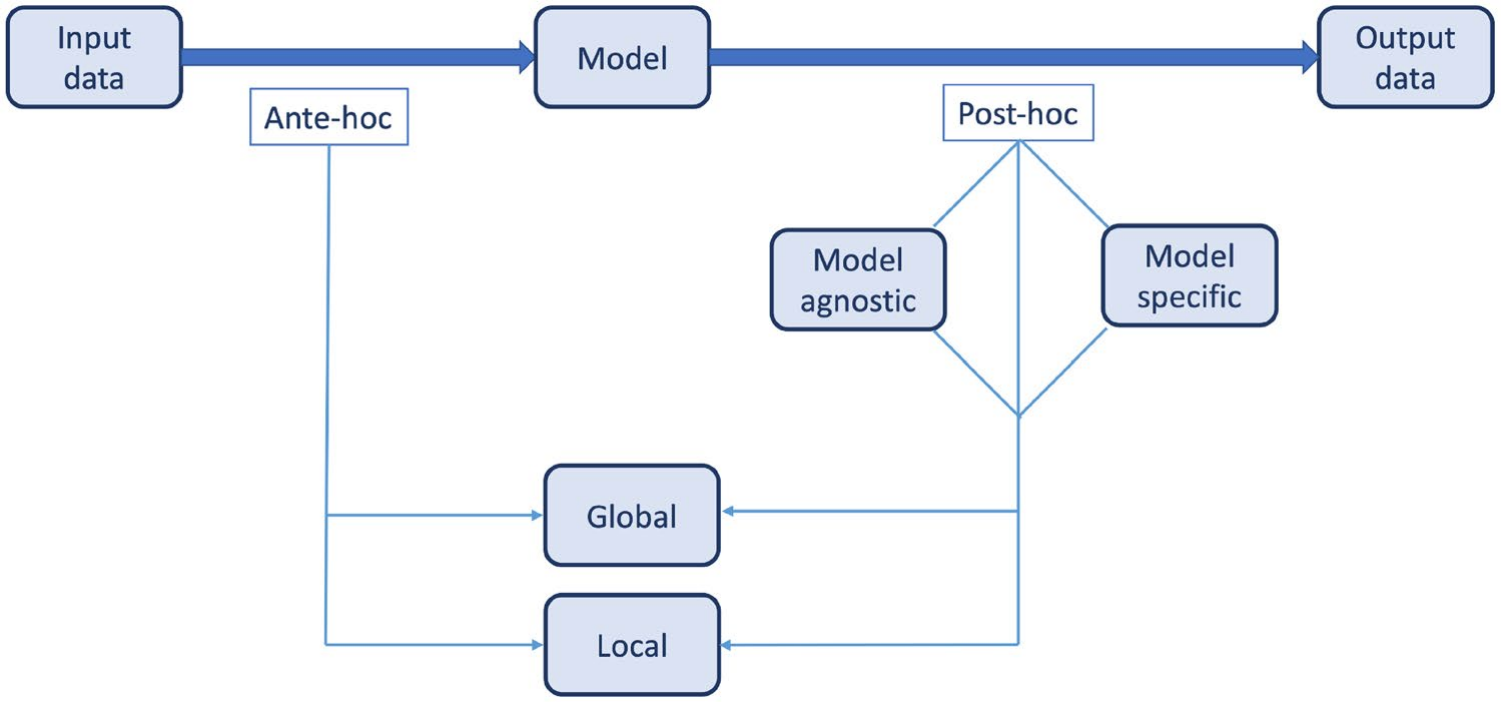
The diagrammatic view of the classification of xAI techniques

Figure 2 summarizes the simplified classification of xAI techniques with a diagrammatic view. (Fig. 2).

Broadly speaking, two types of explainable AI models can be distinguished: post-hoc explainability, occurring after the event in question; and ante-hoc explainability, or so-called, inherent explainability, occurring before the event in question. The concept of xAI can be applied through two approaches: post-hoc and ante-hoc [12]. Post-hoc xAI involves the use of external explainers to interpret a trained model’s behavior during testing. In contrast, ante-hoc xAI incorporates explainability into the AI model's structure from the outset, prioritizing natural understandability while still striving for optimal accuracy during training. Essentially, ante-hoc aims to consider a model’s explainability throughout its development, whereas post-hoc merely explains the model’s behavior after it has been trained [12, 26].

The explainability of machine learning models is generally feasible when models rely on input data that is easily quantifiable and interpretable. There are algorithms, for instance, the decision trees, sparse linear and additive models, or the Bayesian classifiers that are designed with a limited number of internal components, thus allowing the inspection of the model's prediction and/or classification operations. These models provide traceability and transparency in their decision-making [25]. However, in modern AI algorithms, models and data are often complex and high-dimensional, making them difficult to explain with a simple relationship between inputs and outputs. For example, DL models are a category of machine learning algorithms that surrender the model’s understandability for prediction and/or classification accuracy [25]. The DL frameworks are used in applications such as speech and image recognition, natural language processing, and analyzing complex image and sound data. Therefore, explainability techniques for these “black-box” models are post-hoc explainability techniques. First, they resemble DL black-box models into simpler interpretable models, and by doing so, they permit to explore and explain the black-box [12]. These techniques are called xAI, with the main aim to migrate form ***“black-box”*** models into more transparent and interpretable, akin to ***“glass-box”*** models [25]. The scope of an explanation can be either global or local, with global explanations aiming to translate the whole inferential course transparent and intelligible, while locally explainable methods aim to explain individual feature attributions [26].

The common form of post-hoc explainability in medical imaging settings is ***heat maps*** or ***saliency maps***. These maps are a common form of post-hoc explainability that bring out the contribution of each region into the process of decision formation [27]. These methods are not solitary instrumentation available for xAI users, despite their immature state. In the medical imaging field, some other approaches have been already successfully adopted, including methods for *feature visualization* and *prototypical comparisons*. More common general post-hoc explanation methods acceptable for complex medical imaging data embrace the *locally interpretable model-agnostic explanations (LIME)* and *Shapley values (SHAP)*. LIME attempts to understand decisions at the discrete stage by permuting the input sample, while SHAP generates explanations by measuring the contribution of each feature to a specific prediction. LIME and SHAP are generic and applicable to various types of data in healthcare, not limited to medical imaging data. They are commonly used to provide explanations for complex models in the healthcare domain [27, 28].

**Fig. 3:**
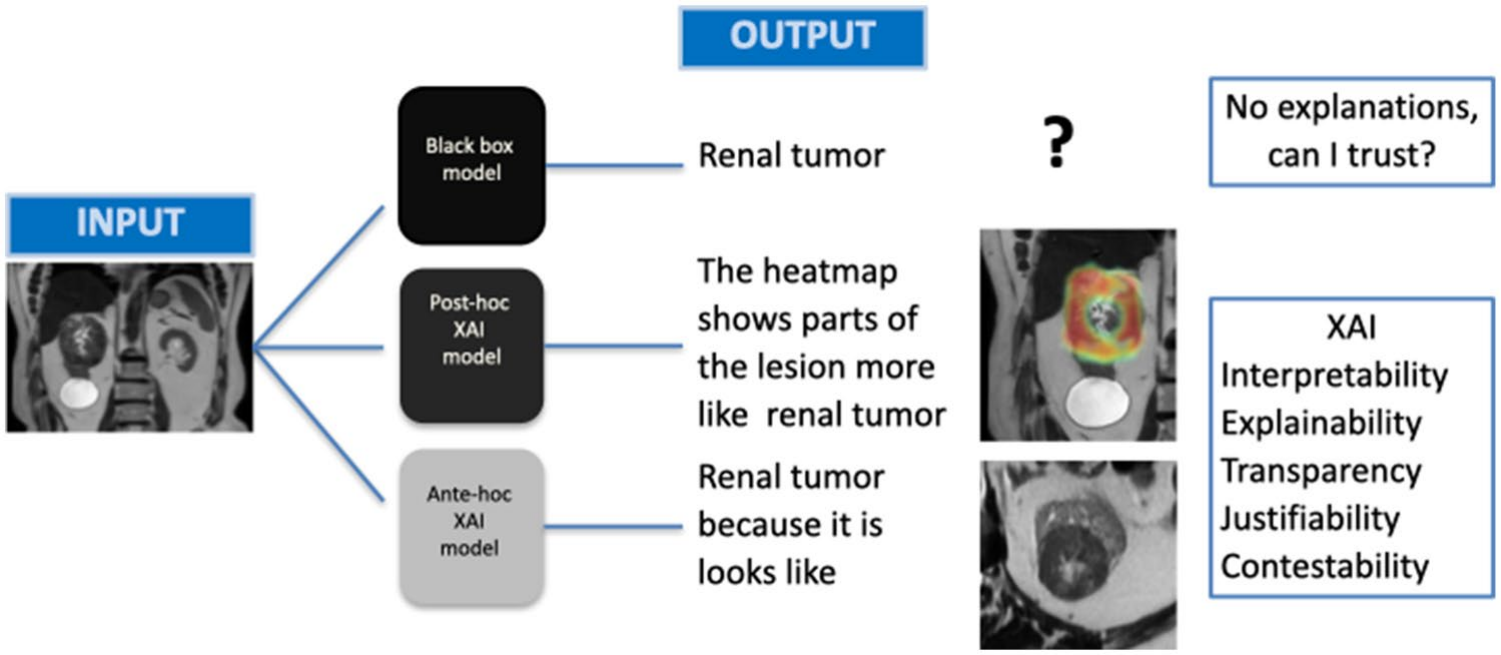
A possible work-flow approach of different Artificial Intelligence (AI) model, applied on a specific input (renal solid mass) including black box, post-hoc Explainable AI (XAI) and ante-hoc XAI. The output of renal tumor is appreciable in all three model but with no explanation on black box model and different approaches of explainability on post-hoc and ante-hoc XAI

Post-hoc xAI methods can be ***model-specific*** or ***model-agnostic*** (Fig. 2). Model-specific methods reshapes DL models in a way to incorporate interpretability context into the structure and learning mechanisms of the model itself, in contrary to model-agnostic methods that operate at the level of the inputs and output of the black box models to handle the explainability issues and to draw explanations. However, ante-hoc methods focus on creating a running model transparent, which is why the ante-hoc methods are intrinsically model-specific. For model-agnostic methods, the internal elements of a model can be ignored, hence these types of models can be applied to any learning approach, while model-specific methods are limited to a determinant subgroup of models [26]. Figure 3 shows possible work-flow approach of different AI model, applied on a specific input (renal solid mass) including black box, post-hoc xAI and ante-hoc xAI.

## Explainable AI in radiology

### Where and how much is explainable AI relevant in radiology

In 2021 an interesting article written by van Leeuwen K.G. and colleagues was published, containing an analysis of 100 commercially available AI radiological products and related scientific evidence [29]. The paper underlines how despite the CE-marked products being analyzed, only a few had related studies on clinical impact (18/100) and, only 36/100 had peer-reviewed efficacy papers published. An aspect that needs a comment on this paper is the transparency aim of the manuscript, despite a clear xAI section is lacking. However, the Authors provide an online up-to-date tool to deepen the approved AI (www.aiforradiology.com), where it is possible to search for a specific AI tool and related information on how it works, its trustworthiness, and its clinical efficacy. From the analysis of this example, one of the main problems concerning AI in radiology and its explainability emerges. In fact, despite the availability of much CE-marked software, having already been released and started to be used, only a few have been analyzed regarding their explainability. The xAI problem could be marginal in the case of AI software that performs individual tasks, such as segmentation or lesion detection, where radiologists have the ability to check and modify the AI output before signing a report. However, in the case of more complex tasks that combine different medical areas and yield results in terms of prognosis or therapeutic strategies, based on different AI approaches, may radiologists be able to critically interpret the output? In this contest, the black-box approach lacks trustworthy and xAI is necessary to assure radiologists, other specialists, and patients the essential tool to merge AI software in real life. Of course, this process is not easy, and it needs time to be assimilated and integrated into the clinical use of AI.

In addition, it is hard to believe that radiologists might have all the knowledge to understand xAI but, some efforts are necessary to acquire some fundamental expertise and principle of xAI to improve the transparency and explainability of AI software that has the potential of decision-making in the medical area. Moving on to a different radiological topic it is possible to make explicit this concept. For instance, not all radiologists know all the Magnetic Resonance Imaging (MRI) functioning or components even if they interpret it for a clinical purpose MRI imaging. However, radiologists that use MRI as a diagnostic tool are aware of the ghosting artifact mechanism, and that allows them not to misinterpret an aortic pulsation on the liver parenchyma as a lesion. Taking this example, it seems important for AI applications in Radiology to have the possibility to understand how outputs are generated to reduce the risk of “dogmatic medicine”, far away from the “evidence-based approach” that drives progress in science now [5, 30].

For that reason, all the available AI tools in the Radiology field, such as segmentation, detection, lesion characterization, and prognosis, need end-users' side attention regarding the artificial neural network data processing accessibility also after the output is obtained. Of course, this extremely challenging task deserves attention and collaboration also from the other actors in the process (data scientists, developers, engineers, product companies, etc..) to improve the xAI process of merging AI tools in clinical practice, by avoiding conscious or unconscious errors that will damage the patient’s health or trust in AI. Nevertheless, pushing xAI to extreme transparency and explainability contains a very complex intrinsic limit. With increasing transparency, interpretability and explainability comes the risk of reducing the performance of these algorithms based on the true deep learning process. Therefore, once the benefits and limitations of xAI in Radiology are clear, we need to start implementing this process on a large scale of users to test the benefits of AI in clinical practice and to adapt the process itself to reality.

An interesting approach to the evaluation of the explainability of an AI system is the one called Z-Inspection; an initiative to assess trustworthy AI in practice [31, 32]. The Z-Inspection procedure has three main phases: (1) *Set Up phase,* during which necessary preconditions are clarified, the team of investigators are defined, the boundaries of the assessment are delineated, and a protocol is created; (2) *Assess phase*, during this phase the use of AI system is inspected, the potential ethical, as well as technical and legal issues are identified, which are further extended to the trustworthy AI ethical values and requirements; (3) *Resolve phase,* this phase engages with the raised issues in the sense of possible ethical tensions, and recommends appropriate procedures.

The adoption of the Z-Inspection process is important to settle on an AI in clinical practice, since it follows the Assessment List for Trustworthy Artificial Intelligence (ALTAI) outlined by the Ethics Guidelines for Trustworthy AI [33].

### Implications of xAI for the radiological profession

Large-scale benefits potentially derivable from AI in medical care are enormous, in terms of process optimization, personalized treatment, and technical implementations, but all these possible scenarios are far from being realized, if possible, drawbacks are not recognized and corrected properly [5]. Being aware of these aspects and realizing the actuality of the thematic is central to preserving the rigor of the medical process as we built it up to now. In fact, AI is taking space not only in the research field but also in clinical practice as mentioned above. In this context, the xAI plays a bridging role in combining the rapid development of AI and its use in practice, in particular in the radiological profession. Thus, being conscious of xAI in the radiological profession implies some changes in the profession itself to avoid a possible catastrophic epilogue such as the one hypothesized by the AI precursor Geoffrey Hinton in 2016: “People should stop training radiologists now. It's just completely obvious that within 5 years deep learning will do better than radiologists.” Luckily, the process of AI implementation has been revealed to be more complex than expected and the role of radiologists is still fundamental; on the other hand, this profession will need implementation and modification to be part of the paradigm shift process. In fact, an important role of radiologists will be, as already happened in the past, to expand their knowledge and merge them with prior expertise. In fact, radiology since its beginning has faced up a wide multitude of technological changes and consequent adaptations that succeeded one other very rapidly, an emblematic example is represented by the X-rays phenomenon described by Roentgen to their clinical application soon after [34]. Therefore, one of the main implications of xAI for radiologists is to keep expanding knowledge in this field to take confidence with this new topic strictly related to medicine and in particular radiology, for improving trustworthiness for them and for patients. In fact, a translational approach is more than ever required in medical disciplines to enhance the benefits of progress and minimize potential drawbacks. Within these considerations, two more aspects need to be highlighted. Firstly, how to use the time obtainable from the automation of certain processes that are currently carried out by the radiologist? Secondly: how to implement knowledge of xAI in radiological practice and during radiology training?

Radiologists’ working schedules will probably evolve in a direction prone to solving more complex cases, where uncertainties or atypical situations make the AI application less performant, or to increase multidisciplinary meetings to merge all the information derived from different AI tools. In fact, as figures are more prone to technology, the role of radiologists in terms of explainability and transparency should be central in the next few years.

In addition, it is emerging how radiologists in training suffer from the lack of adequate training regarding AI [35]. An interesting reflection is provided by Forney et al. [36] regarding how much knowledge is the minimum acceptable for radiologists in training to give them the necessary tools to interpret AI in terms of input and outputs produced. In fact, by doing so, new generations of radiologists will be able to critically assess AI tools and be aware of a large number of biases present in this new entity (e.g., prevalence bias, automation bias, detection bias, negative set bias, etc.). Soon, it will be desirable to assure a basic standardized comprehensive education regarding AI and xAI during the training of radiologists, to prevent a new generation of radiologists from getting lost in the path of integrating AI into their discipline, but on the contrary, to become conscious and critical users of it [37].

### The responsibility of the radiologist

Together with the great hype around the blooming of AI, the role of the radiologist is loaded with additional responsibilities concerning the various steps of the AI workflow. In fact, one of the prerequisites of training AI systems is access to a huge amount of data, in the case of imaging data as ground truth. The first concern regarding the accessibility of these medical data regards data ownership, and informed consent. In fact, it is critical to establish, according to countries’ laws, who is the final owner of this data. Community-dedicated laws are necessary to support physicians in that direction [38]. This aspect is also very sensitive, especially since private companies might use such data to develop AI tools that soon after will generate profit [39]. Strictly linked to data ownership there is another important aspect that radiologists need to know and consider. It regards patients’ privacy and informed consent. In fact, it is essential for privacy protection that data injected into the system are anonymized or pseudo-anonymized to avoid tracing back to individual patients. This aspect is deeply connected with the role of radiologists. Before sharing data, radiologists need to be prepared about which data are trackable or recognizable to a single person, and what is needed to be maintained as data to improve AI system efficiency: examples of data protection are the use of pseudo-anonymized information of patient’s age instead of the more conductible date of birth, or to prefer a system that avoids facial recognition obtainable with a volumetric reconstruction of head and neck [40].

After all these aspects have been managed, another fundamental step needs the radiologists’ attention. In fact, an essential step that assures a good development of AI tools regards the clinical question and consequently the type of data that will be used for training the systems. This will help to reduce potential pitfalls that might affect the AI development and further use of AI tools event if they are built with xAI approach. To reduce biases that might impact outputs, xAI will help radiologists and AI tools developers to choose which data are useful to train the software to solve the clinical question.

Parallel to this, radiologists need also to be aware of data labeling. In fact, careful annotation of imaging data that will be used for training, validation, and testing has a central role in AI tool development. In addition, also the definition of the ground truth deserves important consideration: for some pathologies, in fact, a single radiological modality is sufficient to define the diagnosis (e.g., pulmonary CT for pneumothorax) while, some other abnormal entities need a different images modality, imaging follow-up or support from other specialties (e.g., atypical pneumonia to confirm with a second CT after medical therapy). This issue intrinsically contains the risk of weakening the AI training due to the image findings are not directly sufficient, in real life, for the final diagnosis and so, the risk of higher uncertainty for the algorithms or error is high [39].

Another important responsibility of radiologists is transparency with both AI solution developers and patients: xAI, in fact, will support these aspects that will improve trustworthiness and will improve the use of AI in a clinical setting. With transparency, another crucial aspect needs consideration: the responsibility of the medical diagnosis. A large debate is present about the final responsibility of AI tools, but the main direction is prone to consider the radiologist that uses the AI-support as the final responsible [11, 39]. Of course, this aspect is more acceptable with xAI than with black box, and lots of steps need to be taken to consolidate this position and ensure protection for both radiologists and patients.

All the consideration above-mentioned are important to reduce the possibility of pitfalls in the use of AI tool in radiology, even if it is xAI. Another aspect to be considered is actual limit of xAI that cannot be applied at the moment on every AI tool and that explainability is not coincident with high level-decision in every approach in medical practice. Radiologists need to be aware of these limitations to avoid potential biases in the xAI usage [27].

Finally, the most important responsibility for radiologists, encompassing all the others, is to remain critical of the software itself, AI developers, and all the users. In fact, only with constructive critical collaboration among all the professional figures and patients, it will be possible to improve the comprehension of the benefits and limits of AI on specific tools [41].

## Recommendations to adopt explainable AI in radiology

- The incorporation of xAI algorithms and the inclusion of explanatory components should be carefully considered in the development of any high-risk AI system to be used in healthcare and particularly in radiological practice.
- Patients’ informed consent should be built in the clearest and most intelligible manner considering as many as possible xAI concepts including *Interpretability, Explainabiliy, Transparency, Justifiability, and Contestability.*
- Anonymization or pseudo-anonymization of patient data is of utmost importance to comply with current EU regulations. The radiologist can play a key role in making all parties aware of both the importance of sharing data for building biobanks to train artificial intelligence models and the protection of sensitive information.
- Data ownership should be carefully considered before sharing data. This aspect includes important ethical considerations if a profit corporation is involved in the process. Transparency of the entire process is expected to improve the trustworthiness of both medical end-users and patients.
- The radiologist as an end user of xAI, should be aware of the current limitations of xAI in relation to individual decisions, where xAI shows scarce illumination, compare to those explanations applied to global AI processes, such as model development and knowledge discovery.
- Radiologists should be aware of the advent of xAI and the radiological academic community should also take care of the dissemination of the basic concepts of xAI among radiologists, residents, and medical students.
- Constructive critical communication of all xAI processes should be encouraged among all the professional figures involved and, when necessary, with patients.

## Conclusions

The AI has already stepped either in the scientific reality or the quotidian life of the radiologist with the huge success. However, there is still lack of understanding of xAI and its incorporation into the real world of the Radiologists, although the increasing focus on incorporating ethical standards into AI technology design and implementation. The SIRM introduces an xAI-white paper, which is intended to aid Radiologists, medical practitioners, and scientists. We provided an overview of the emerging field of xAI, the black-box problem behind the success of the AI and the xAI methods to unveil the black-box into a glass-box. We stated the role, and responsibilities of the Radiologists for appropriate use of the AI-technology, how it is relevant in the radiology field and finally, we provided some recommendations to adopt explainable AI in the radiology practice.
